# Atherosclerotic plaque vulnerability is increased in mouse model of lupus

**DOI:** 10.1038/s41598-020-74579-8

**Published:** 2020-10-27

**Authors:** Marie-Laure Santiago-Raber, Fabrizio Montecucco, Nicolas Vuilleumier, Kapka Miteva, Daniela Baptista, Federico Carbone, Sabrina Pagano, Aline Roth, Fabienne Burger, Francois Mach, Karim J. Brandt

**Affiliations:** 1 Department of Pathology and Immunology, Faculty of Medicine, University of Geneva, Geneva, Switzerland; 2 Department of Genetic Medicine, Laboratory and Pathology, Geneva University Hospitals, Geneva, Switzerland; 3 Division of Laboratory Medicine, Faculty of Medicine, University of Geneva, Geneva, Switzerland; 4 Ospedale Policlinico San Martino Genoa–Italian Cardiovascular Network, 10 Largo Benzi, 16132 Genoa, Italy; 5 First Clinic of Internal Medicine, Department of Internal Medicine and Centre of Excellence for Biomedical Research (CEBR), University of Genoa, 6 viale Benedetto XV, 16132 Genoa, Italy; 6 Division of Cardiology, Foundation for Medical Researches, Department of Medicine Specialties, Faculty of Medicine, University of Geneva, Av. de la Roseraie 64, 1211 Geneva 4, Switzerland; 7 Present Address: AMAL Therapeutics, Av. de la Roseraie 64, 1211 Geneva 4, Switzerland

**Keywords:** Atherosclerosis, Atherosclerosis, Systemic lupus erythematosus, Systemic lupus erythematosus

## Abstract

Anti-apolipoprotein A-1 (anti-apoA-1 IgG) and anti-double stranded DNA (anti-dsDNA IgG) autoantibodies have been described as mediators of atherogenesis in mice and humans. In the present study, we aim to investigate the association between atherosclerotic parameters, autoantibodies and plaque vulnerability in the context of systemic lupus erythematosus (SLE). We therefore bred a lupus prone-mouse model (Nba2.*Yaa* mice) with Apoe^−/−^ mice resulting in Apoe^−/−^Nba2.*Yaa* mice spontaneously producing anti-apoA-1 IgG antibodies. Although Apoe^−/−^Nba2.*Yaa* and Apoe^−/−^ mice subject to a high cholesterol diet displayed similar atherosclerosis lesions size in aortic roots and abdominal aorta, the levels of macrophage and neutrophil infiltration**,** collagen, MMP-8 and MMP-9 and pro-MMP-9 expression in Apoe^−/−^Nba2.*Yaa* mice indicated features of atherosclerotic plaque vulnerability. Even though Apoe^−/−^Nba2.*Yaa* mice and Apoe^−/−^ mice had similar lipid levels, Apoe^−/−^Nba2.*Yaa* mice showed higher anti-apoA-1 and anti-dsDNA IgG levels. Apoe^−/−^Nba2.*Yaa* mice displayed a reduction of the size of the kidney, splenomegaly and lymph nodes (LN) hypertrophy. In addition, anti-apoA-1 and anti-dsDNA IgG increased also in relation with mRNA levels of GATA3, IL-4, Bcl-6 and CD20 in the spleen and aortic arch of Apoe^−/−^Nba2.*Yaa* mice. Our data show that although atherosclerosis-lupus-prone Apoe^−/−^Nba2.*Yaa* mice did not exhibit exacerbated atherosclerotic lesion size, they did show features of atherosclerotic plaque destabilization in correlation with the increase of pro-atherogenic autoantibodies.

## Introduction

The prevalence of cardiovascular (CV) events in patients with autoimmune diseases such as systemic lupus (SLE) is higher than in the general population^1,2^. During the last decade, a better understanding of the genetic and biological mechanisms underlying autoimmunity-related CVD risk has emerged^1–3^. In the complex context of genetic, environmental and potential microbiota-related factors, autoantibodies have been recognized as important modulators of vascular inflammation and atherogenesis^4–8^. Nevertheless, the function of humoral autoimmunity on atherogenesis and/or related to CV risk is complex. Humoral immunity is dependent on B and T cells, and particularly of follicular helper T cells (T_FH_) that are mainly characterized by the expression of the master transcriptional factor B cell lymphoma 6 (Bcl-6)^9^. In this context, the expression of Bcl-6 in circulating T_FH_ cells positively correlates with the level of disease activity in SLE^10,11^.

Among pro-atherogenic autoantibodies potentially relevant for CV risk, autoantibodies against apolipoprotein A-1 (anti-apoA-1 IgG), the principal protein component of high-density lipoprotein (HDL), have gained substantial interest. Initially discovered in 1998 by Dinu and colleagues in patients with SLE, anti-apoA-1 IgG were found to be associated with a higher prevalence and incidence of coronary artery disease (CAD), independently of traditional CV risk factors in autoimmune and non-autoimmune settings^6,12–16^. According to the preclinical autoimmunity concept, the presence of circulating autoantibodies can be detected a long time before the clinical manifestation and associated with a poorer prognosis^17,18^. We recently demonstrated that anti-apoA-1 IgGs are present in up to 20% of the whole population and are independently associated with higher prevalence and incidence of CAD^19–22^. In vivo and in vitro studies have demonstrated that anti-apoA-1 IgG antibodies have pro-inflammatory, pro-arrhythmogenic and pro-thrombotic properties. Furthermore, anti-apoA-1 IgG antibodies promote atherogenesis, myocardial necrosis and death in mice by a TLR2/TLR4/CD14 dependent process^19–22^. Approximately 20% and 13% of nonautoimmune or SLE patients, respectively, with acute coronary syndrome were found to have circulating anti­apoA­I antibodies, compared with only 1% of healthy individuals^23^. The lupus-prone mouse model used in the study was C57BL/6 mice congenic for New Zealand black (NZB) autoimmunity 2 (Nba2) locus on chromosome 1, that peaked at the FcgR2b gene encoding FcgammaRIIB. Nba2 is a major lupus susceptibility locus derived from the lupus-prone strain NZB. In addition, in Nba2.*Yaa* lupus-prone mice, the males bear the Y-linked autoimmune acceleration (*Yaa*) mutation that originates from the lupus-prone mouse strain BXSB and confers accelerated autoimmunity^24^. *Yaa*-bearing mice have an X-linked *tlr7* gene duplication on the Y chromosome^25^. These mice develop an autoreactive B cell response to RNA-related antigens due to TLR7 gene duplication, which results in high levels of nucleolar-related autoantibodies and splenomegaly. Also, Nba2 male mice bearing the *Yaa* mutation develop significantly increased percentages of circulating monocytes in parallel to the development of lupus-like autoimmune manifestations^26^. Furthermore, dead cells in atherosclerosis promote the release of extracellular dsDNA, found in human atherosclerotic lesions^27^, the levels of which correlate with the risk of CV events^28^. Moreover, the generation of anti-dsDNA antibodies critically aggravates atherosclerosis lesion formation^29^. Despite the achieved improvement of first year survival, the late peak of mortality in SLE, largely due to CV diseases, has remained almost unchanged^30^. In order to reveal the mechanism leading to higher CV mortality in SLE, we investigated the association between autoantibodies, atherosclerotic parameters and plaque vulnerability in the context of SLE. To address this issue, we crossed the lupus-prone Nba2.*Yaa* mouse model with atherosclerosis-prone Apoe^−/−^ mice, thus generating a mouse model that enabled the study in vivo of the potential relation between autoantibodies, atherosclerotic plaque vulnerability, lymphocyte polarization and lipid profile.

## Results

### Atherosclerosis-lupus-prone mice develop vulnerable atherosclerotic plaques

**Figure 1:**
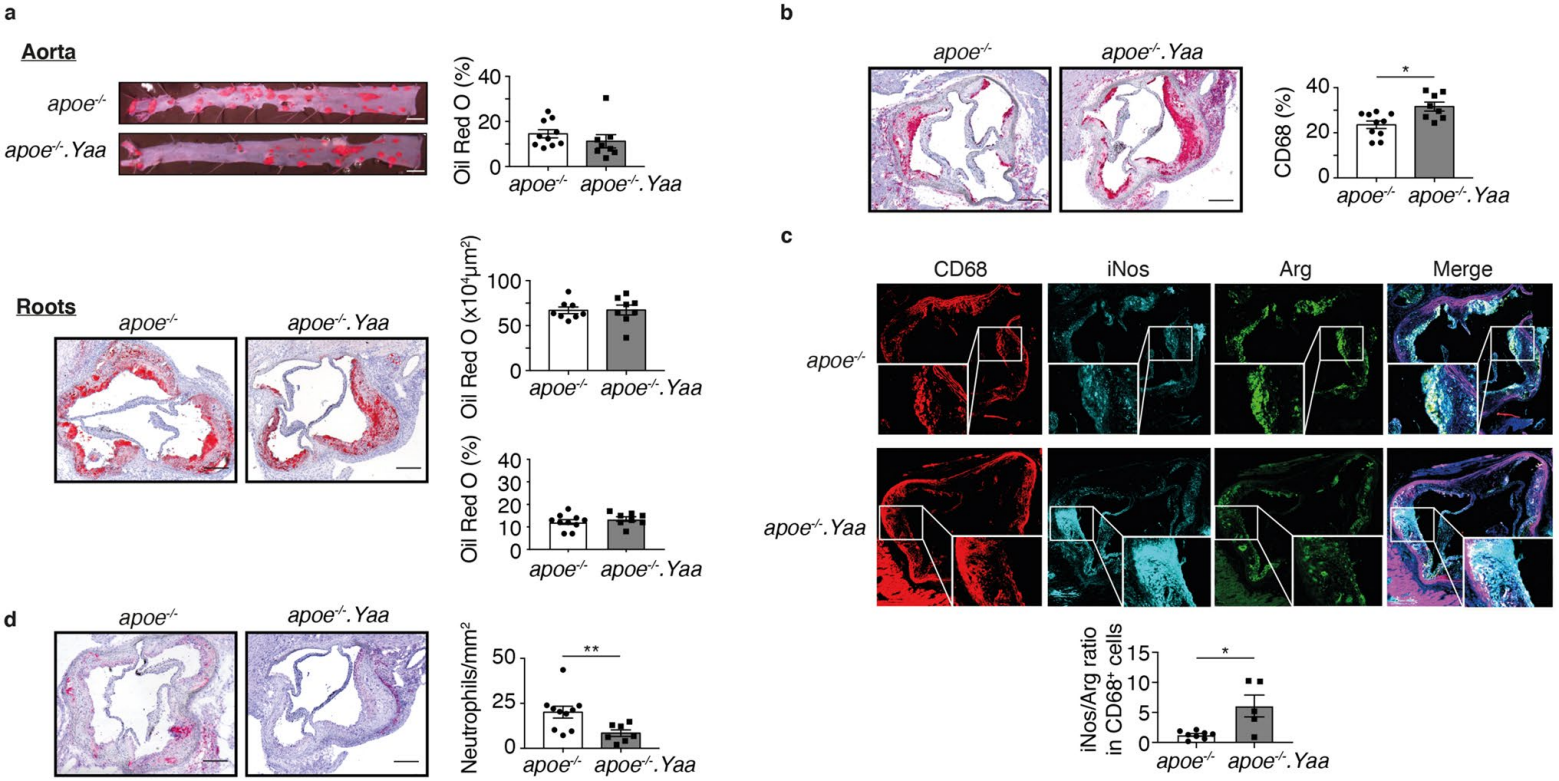
Nba2.*Yaa* mutation in Apoe^−/−^mice does not affect atherosclerosis lesion size. (**a**) Representative pictures of Oil Red O stained atherosclerotic lesions in the abdominal aorta and roots of Apoe^−/−^ or Apoe^−/−^Nba2.*Yaa* (*apoe*^*−/−*^*.Yaa*) mice. Bar graphs represent the median ± SEM of Oil Red O quantification of atherosclerotic lesion size, expressed as % of total area and as μm^2^ in Apoe^−/−^ or Apoe^−/−^Nba2.*Yaa* mice on HCD (n = 8–10 mice/group). (**b**) Representative pictures and quantification of CD68 staining in the roots of Apoe^−/−^ or Apoe^−/−^Nba2.*Yaa* mice on HCD. Bar graphs represent the median ± SEM of CD68 cell quantification, expressed as % of total area in the roots of Apoe^−/−^ or Apoe^−/−^Nba2.*Yaa* mice on HCD ( n = 8–10 mice/group). (**c**) Representative pictures and ratio of quantification of CD68, iNos and Arginase (Arg) staining in the roots of Apoe^−/−^ or Apoe^−/−^Nba2.*Yaa* mice on HCD. Bar graphs represent the median ± SEM of iNos/Arg ratio of quantification relative to total area of the roots of Apoe^−/−^ or Apoe^−/−^Nba2.*Yaa* mice on HCD (n = 8–10 mice/group). (**d**) Representative pictures of neutrophils staining in the roots of Apoe^−/−^ or Apoe^−/−^Nba2.*Yaa* mice on HCD. Bar graphs represent the median ± SEM of neutrophil quantification, expressed as mm^2^ in the roots of Apoe^−/−^ or Apoe^−/−^Nba2.*Yaa* mice on HCD (n = 8–10 mice/group) and **p < 0.01. Original magnification, × 10. Scale bars, 400 μm. The nonparametric Mann–Whitney U test was used for statistical analysis: *p ≤ 0.05; **p ≤ 0.005. All data were represented as mean ± sem.

**Figure 2:**
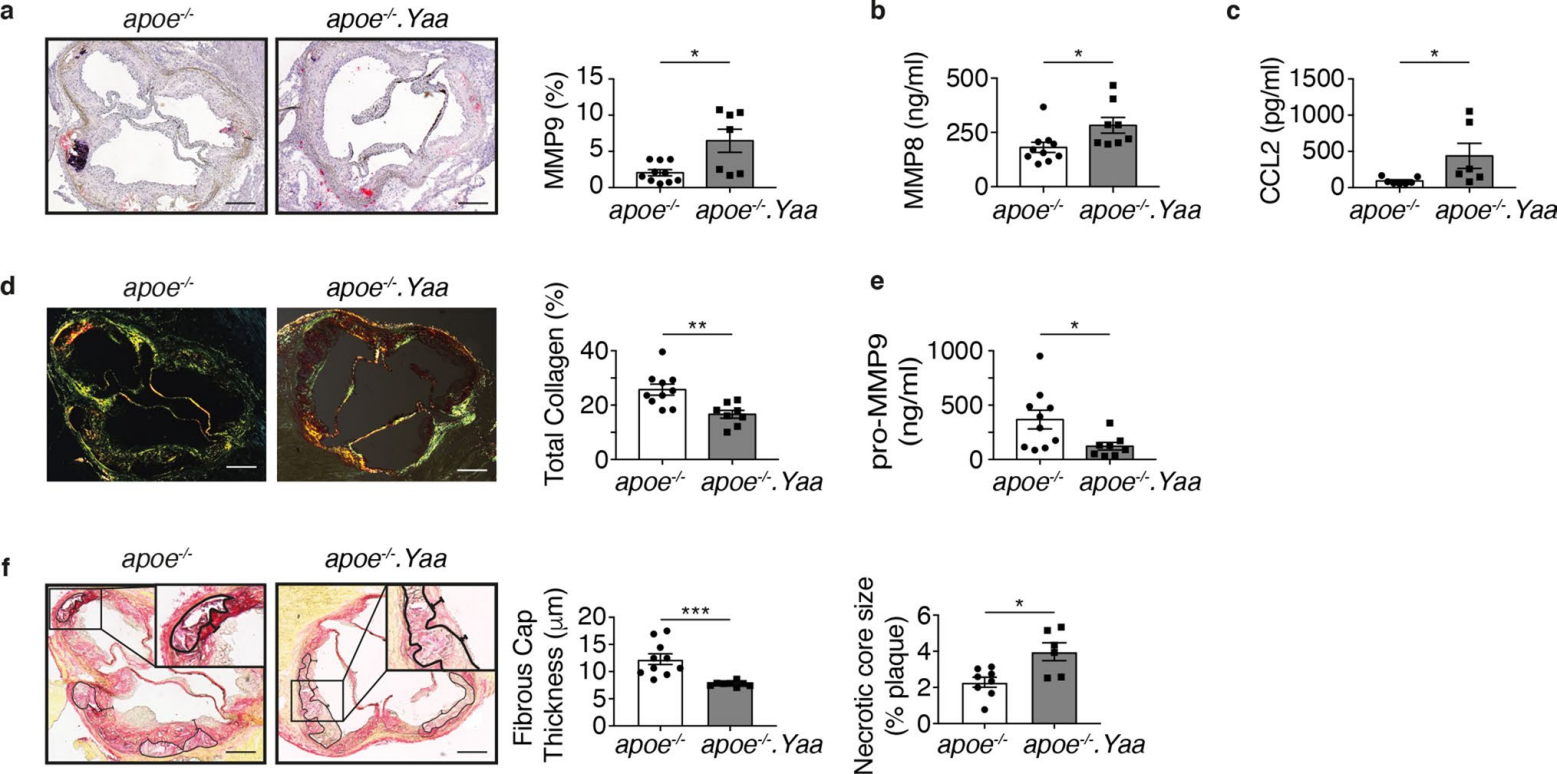
Apoe^−/−^Nba2.*Yaa* (*apoe*^*−/−*^*.Yaa*) mice on a high cholesterol diet develop vulnerable atherosclerotic plaques. Bar graphs represent the median ± sem of (**a**) Representative pictures and quantification of MMP-9 expressed as % of total roots area. (**b**) MMP-8 quantification in the serum (**c**) CCL2 quantification in the serum, (**d**) picrosirius red staining of total collagen in the roots, expressed as % of total roots area, and (**e**) pro-MMP-9 quantification in the serum of Apoe^−/−^ or Apoe^−/−^Nba2.*Yaa* mice on HCD (n = 8–10 mice/group). (**f**) Representative pictures and quantification of fibrous cap thickness (μm) and necrotic core size expressed as % of total roots area. (n = 8–10 mice/group). Original magnification,  ×10. Scale bars, 400 μm. The nonparametric Mann–Whitney U test was used for statistical analysis: *p ≤ 0.05; **p ≤ 0.005. Data were represented as mean ± sem.

In the advanced atherosclerotic model, 11-week-old Apoe^−/−^ and Apoe^−/−^Nba2.*Yaa* mice were fed HCD for 11 weeks. Although the initial weight gain of Apoe^−/−^Nba2.*Yaa* mice was lower than for Apoe^−/−^ mice, both groups gained weight as expected under HCD (Table 1). The investigated laboratory parameters showed that Apoe^−/−^Nba2.*Yaa* mice suffered from thrombocytopenia compared to Apoe^−/−^ mice (Table 1). The size of atherosclerotic lesions in the abdominal aorta and in the aortic roots was similar between Apoe^−/−^Nba2.*Yaa* and Apoe^−/−^ mice (Fig. 1a). However, the macrophages in aortic roots increased in Apoe^−/−^Nba2.*Yaa* mice compared to Apoe^−/−^ mice. Furthermore, the ratio between iNos (M1 macrophage) and Arginase (M2 Macrophage) in CD68^+^ cells increased in Apoe^−/−^Nba2.*Yaa* compared to Apoe^−/−^ mice (Fig. 1c). Finally, the number of neutrophils decreased in Apoe^−/−^Nba2.*Yaa* (Fig. 1d). Apoe^−/−^Nba2.*Yaa* mice showed induction of plaque vulnerability parameters associated with destabilization of the plaques (Fig. 2). In this context, we observed that MMP-9, MMP-8 and CCL2 in aortic roots increased (Fig. 2a–c), whereas the level of collagen and serum pro-MMP-9 in aortic roots decreased (Fig. 2d,e). We also noted that the fibrous cap thickness decreased and the size of necrotic core increased in Apoe^−/−^Nba2.*Yaa* mice in comparison with Apoe^−/−^ mice (Fig. 2f). These data suggest that the generated atherosclerosis-lupus-prone Apoe^−/−^Nba2.*Yaa* mice have a higher state of plaque vulnerability despite atherosclerotic plaque sizes comparable to those of Apoe^−/−^mice.

**Table 1:** Clinical and laboratory parameters at sacrifice of adult ApoE^−/−^ mice after 11 weeks of high-cholesterol diet.

Mouse profile	*apoe*^*−/−*^ (n = 10)	*apoe*^*−/−*^*.Yaa* (n = 8)	*p*-value
**Clinical features**			
Body weight at baseline (g)	26.3 (25.3–30.0)	24.4 (24.1–25.2)	**0.037**
Body weight at month 1 (g)	28.0 (25.3–31.0)	25.7 (25.5–26.1)	0.245
Body weight at month 2 (g)	30.3 (28.4–31.8)	27.3 (26.5–28.1)	**0.003**
Body weight at sacrifice (g)	35.6 (34.9–36.7)	30.7 (29.6–31.6)	**0.001**
Lymph node weight (mg)	99 (77–158)	235 (185–290)	**0.003**
**Biochemical**			
Total-c (mmol/l)	31.5 (25.1–36.0)	23.9 (18.1–29.2)	0.120
LDL-c (mmol/l)	27.8 (21.8–32.3)	19.1 (14.8–23.9)	0.083
HDL-c (mmol/l)	10.7 (9.0–12.0)	8.6 (5.6–9.4)	0.120
TAG (mmol/l)	1.1 (0.8–1.8)	1.2 (0.9–1.8)	0.859
FFA (mmol/l)	1.4 (1.2–1.7)	1.5 (1.4–1.7)	0.450
Glycaemia (mmol/l)	15.9 (12.2–19.3)	16.1 (14.7–18.8)	0.625
**Haematology**			
Hb (g/dl)	10.7 (10.3–11.3)	10.0 (8.7–11.7)	0.477
RBC count (×10^6^/μl)	7.6 (7.0–7.9)	6.5 (5.6–7.7)	0.100
WBC count (×10^3^/μl)	4.8 (3.7–8.0)	3.6 (1.6–4.9)	0.138
Lymphocytes (×10^3^/μl)	3.1 (0.9–6.9)	2.1 (1.3–3.8)	0.832
PLT (×10^3^/μl)	1159 (937–1328)	541 (305–813)	**0.005**
**Inflammation**			
MPO (ng/ml)	199.8 (164.8–265)	239.15 (200.15–347.02)	0.564
TIMP-1 (ng/ml)	2.59 (1.66–2.95)	2.65 (1.77–3.80)	0.564
CXCL1 (pg/ml)	238.64 (113.56–388.55)	193.43 (171.56–341.79)	1.000

Data are expressed as median (interquartile range).

Bold values correspond to significant *p* values.

total-c: total cholesterol; LDL-c: low-density lipoprotein cholesterol; HDL-c: high-density lipoprotein cholesterol; TAG: triglyceride; FFA: free fatty acids; Hb: haemoglobin; RBC: red blood cell; WBC: white blood cells; PLT: platelet; MPO: myeloperoxidase; TIMP: tissue inhibitor of matrix metalloproteinase; CXCL: (C-X-C motif) ligand.

*p*-value calculated according to Mann–Whitney U test.

### Apoe^−/−^Nba2.*Yaa* mice have elevated autoantibodies, small kidney, splenomegaly and lymph node hypertrophy

**Figure 3:**
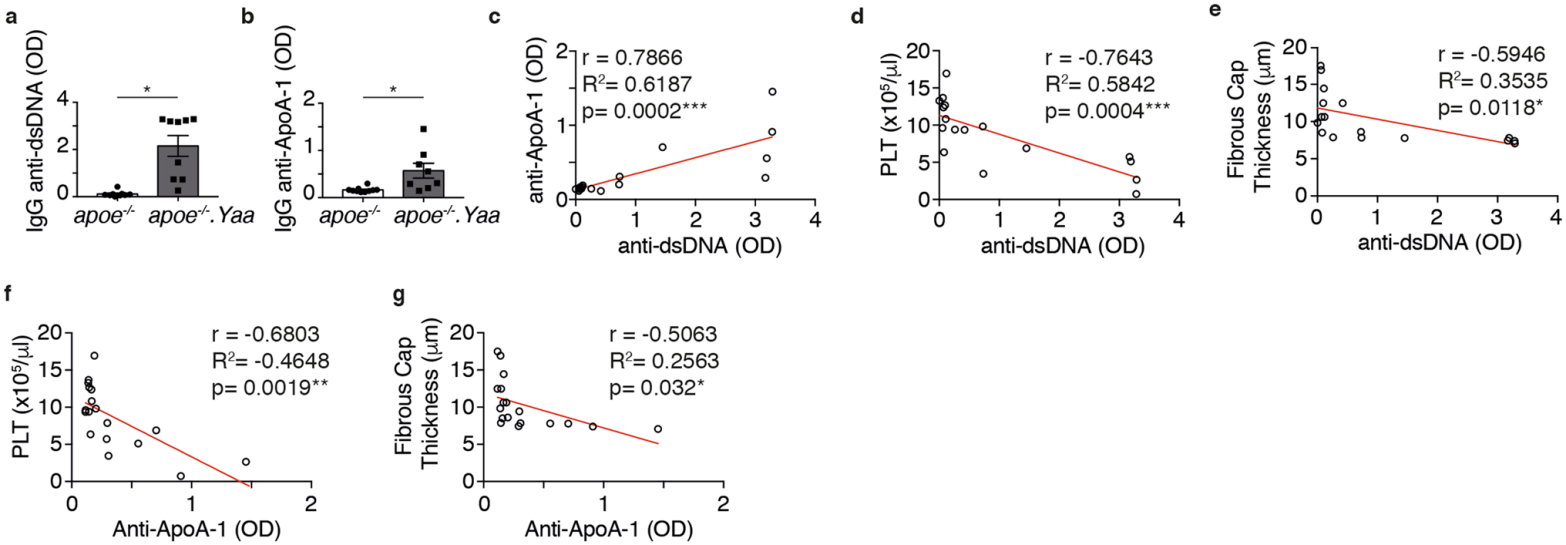
Apoe^−/−^Nba2.*Yaa* (*apoe*^*−/−*^*.Yaa*) mice exhibit elevated anti-dsDNA and anti-ApoA-1 circulating antibodies. Bar graphs represent the median ± s.e.m. of (**a**) anti-dsDNA IgG and (**b**) anti-ApoA-1 IgG autoantibody quantification in the serum, measured as optical density (OD) in Apoe^−/−^ or Apoe^−/−^Nba2.*Yaa* mice on HCD (n = 8–10 mice/group). The nonparametric Mann–Whitney U test was used for statistical analysis: *p ≤ 0.05. Spearman’s rank correlation coefficients between anti-dsDNA IgG and anti-ApoA-1 IgG (**c**), PLT (**d**), and Fibrous cap thickness (**e**). Spearman’s rank correlation coefficients between anti-ApoA-1 IgG and PLT (**f**) and Fibrous cap thickness (**g**).

**Figure 4:**
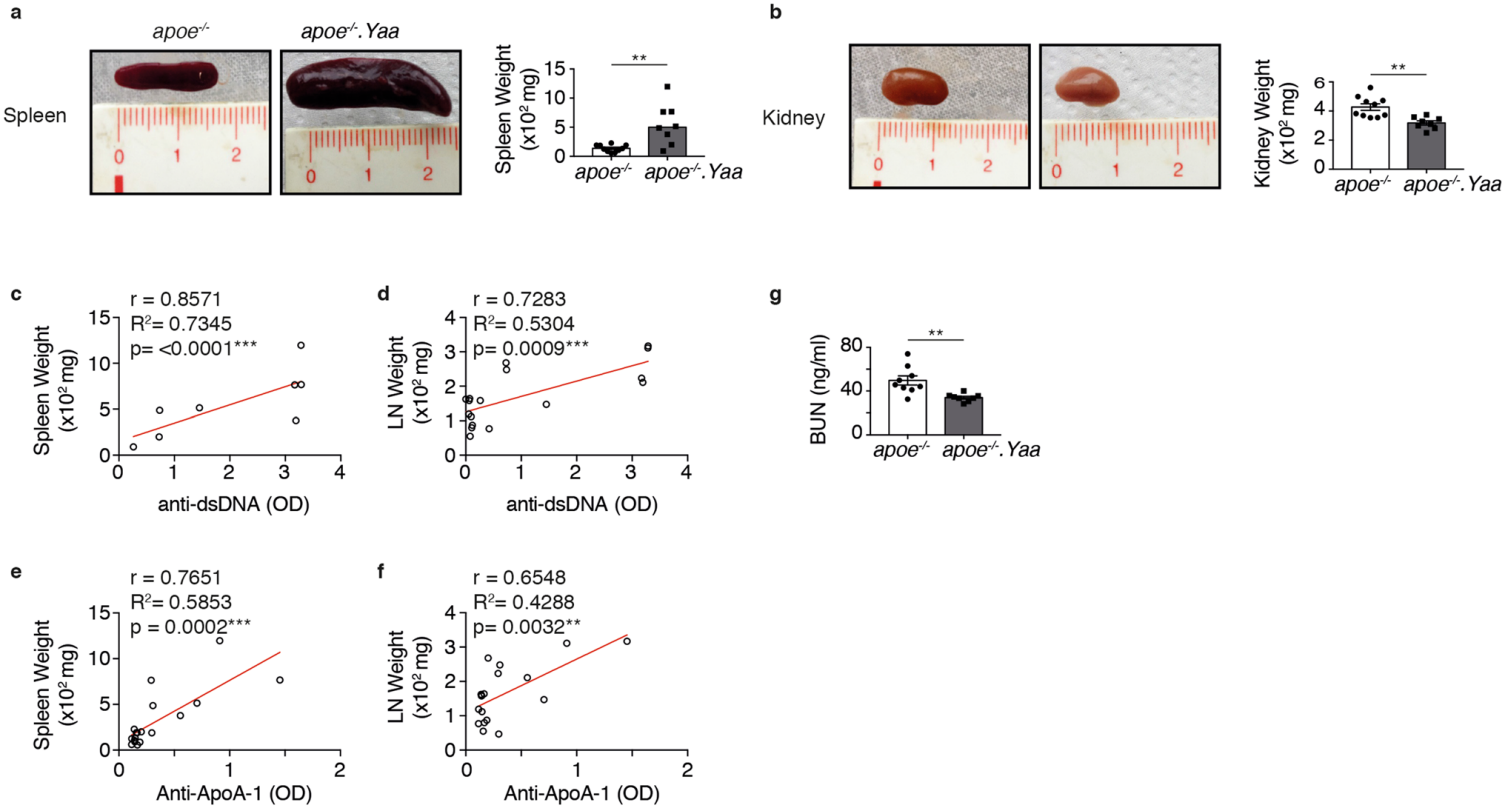
Apoe^−/−^Nba2.*Yaa* mice develop small kidney and splenomegaly. (**a**) Representative pictures of spleen size and weight quantification in Apoe^−/−^ or Apoe^−/−^Nba2.*Yaa* (*apoe*^*−/−*^*.Yaa*) mice. Bar graph represents the median ± s.e.m. of spleen weight quantification in Apoe^−/−^ or Apoe^−/−^Nba2.*Yaa* mice on HCD. (**b**) Representative pictures of kidney size and weight quantification in Apoe^−/−^ or Apoe^−/−^Nba2.*Yaa* mice. Bar graph represents the median ± s.e.m. of kidney weight quantification. Spearman’s rank correlation coefficients between anti-dsDNA IgG and spleen weight (**c**) or LN weight (**d**). Spearman’s rank correlation coefficients between anti-ApoA-1 IgG and spleen weight (**e**) or LN weight (**f**). (**g**) Quantification of serum blood urea nitrogen (BUN) measurement in Apoe^−/−^ or Apoe^−/−^Nba2.*Yaa* mice on HCD (n = 8–10 mice/group). The nonparametric Mann–Whitney U test was used for statistical analysis: *p ≤ 0.05; **p ≤ 0.005.

To examine whether the Nba2.*Yaa* mutation in Apoe^−/−^ mice affects autoantibody production, 11-week-old Apoe^−/−^ and Apoe^−/−^Nba2.*Yaa* mice were subjected to HCD for 11 weeks and serum was collected and tested for antibodies against dsDNA and ApoA-1 by ELISA. Levels of anti-dsDNA and anti-ApoA-1 IgG were significantly higher in Apoe^−/−^Nba2.*Yaa* mice compared with Apoe^−/−^ mice (Fig. 3a,b). Furthermore, the level of increase for both autoantibodies was correlated (Fig. 3c). Consistently with the vulnerable parameters observed in Apoe^−/−^Nba2.*Yaa* mice, PLT and fibrous cap thickness were inversely correlated with the level of anti-dsDNA IgG (Fig. 3d,e). As anti-dsDNA and anti-ApoA-1 IgG production are associated, we observed the same results with PLT, fibrous cap thickness and anti-ApoA-1 IgG level (Fig. 3f,g). Interestingly, the weight of the spleen and lymph nodes increased significantly, while that of the kidney decreased in Apoe^−/−^Nba2.*Yaa* mice versus Apoe^−/−^ mice (Fig. 4a,b and Table 1). A correlation between autoantibody titers and the weight of the spleen and lymph nodes was also observed (Fig. 4c,d). However, assessment of kidney function did not suggest the existence of a significant renal dysfunction in Apoe^−/−^Nba2.*Yaa* mice compared with Apoe^−/−^ mice, as indicated by the measurement of blood urea nitrogen (BUN) (Fig. 4g). This result is consistent with the negative staining for IgG and IgM deposition in frozen kidney sections (data not shown) and the fact that the Nba2.*Yaa* mouse model required at least 12 months to fully develop glomerulonephritis.

### Apoe^−/−^Nba2.*Yaa* mice exhibit Th2, T_FH_ and B cell mRNA markers

**Figure 5:**
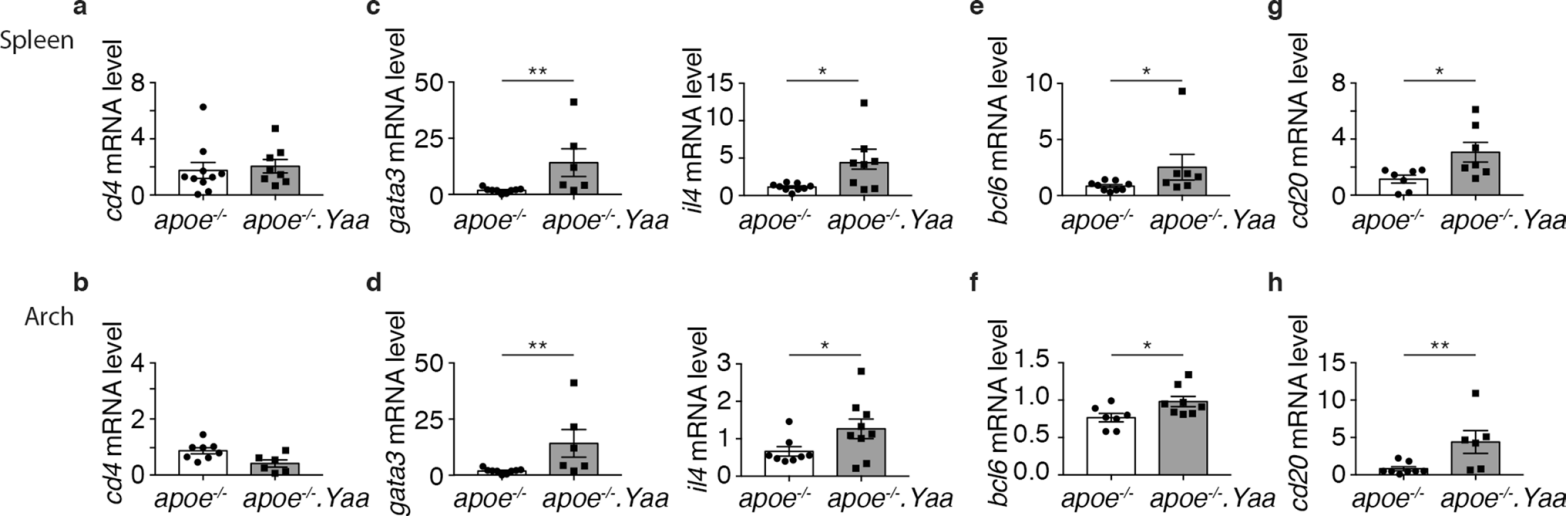
Apoe^−/−^Nba2.*Yaa* mice exhibit Th2, T_FH_ and B cell mRNA markers in the spleen and the aortic arch in HCD. Bar graphs represent the median ± s.e.m. of mRNA quantification of (**a**) *cd4* expression in the spleen, (**b**) *cd4* expression in the arch, **(c)** *gata3 and il4* expression in the spleen, (**d**) *gata3 and il4* expression in the arch, (**e**) *bcl6* expression in the spleen, (**f**) *bcl6* expression in the arch, (**g**) *cd20* expression in the spleen and (**h**) *cd20* expression in the arch in Apoe^−/−^ or Apoe^−/−^Nba2.*Yaa* mice on HCD (n = 8–10 mice/group). The nonparametric Mann–Whitney U test was used for statistical analysis: *p ≤ 0.05; **p ≤ 0.005..

Taking into account that SLE is a systemic autoimmune disease, T and B cell subsets were quantified in the secondary lymphoid organs and aortic roots after 11 weeks of HCD. While CD4 mRNA levels were not affected either in the spleen or in the aortic arch of Apoe^−/−^Nba2.*Yaa* mice compared with Apoe^−/−^ mice (Fig. 5a,b), *gata3* and *il4* mRNA expression was significantly increased both in the spleen and aortic arch (Fig. 5c,d). mRNA expression of Th2 marker *gata3* was significantly upregulated while *il10* increased in the aortic arch of Apoe^−/−^Nba2.*Yaa* mice compared with Apoe^−/−^ mice in advanced atherosclerosis (Table 2). *il10* mRNA expression in the spleen was, however, significantly downregulated in Apoe^−/−^Nba2.*Yaa* mice versus Apoe^−/−^ mice on HCD, whereas it was similar in the lymph nodes of both mice strains (Table 3). In parallel the gene expression of Th1, associated gene tim3 prominently increased in the spleen of Apoe^−/−^Nba2.*Yaa* mice versus Apoe^−/−^ mice on HCD. *bcl*6 mRNA was upregulated in the spleen and aortic arch of Apoe^−/−^Nba2.*Yaa* mice compared with Apoe^−/−^ mice on HCD (Fig. 5e,f). Consistently, *cd20* mRNA gene expression was upregulated both in the spleen and aortic arch of Apoe^−/−^Nba2.*Yaa* mice in comparison with Apoe^−/−^ mice in advanced atherosclerosis (Fig. 5g,h). In conclusion, our results suggest that the Nba2.*Yaa* mutation in Apoe^−/−^ mice leads to the induction of mRNA markers of Th2 and T_FH_ cell populations, which in turn stimulate the induction of CD20^+^ B cells in Apoe^−/−^Nba2.*Yaa* mice on HCD.

**Table 2:** Expression of B cell, T cell and inflammatory markers in aortic arches.

Marker (mRNA fold increase)	*apoe*^*−/−*^ (n = 10)	*apoe*^*−/−*^*.Yaa* (n = 8)	*p*-value
**Aortic arches**			
Th1			
*tim3*	0.83 (0.62–2.14)	2.29 (0.56–2.58)	0.364
*ifnγ*	0.90 (0.35–3.27)	0.06 (0.00–1.05)	0.343
Treg			
*foxp3*	1.03 (0.80–1.25)	6.02 (2.36–25.33)	0.071
*il10*	0.98 (0.80–1.17)	2.00 (1.77–3.20)	0.051
Th17			
*rorc*	1.60 (0.75–2.95)	0.89 (0.54–1.58)	0.407
*il17*	0.83 (0.48–1.56)	0.84 (0.34–3.01)	1.000
*cxcl1*	1.17 (0.45–1.53)	0.54 (0.30–3.35)	0.740
*ccl2*	1.46 (0.83–1.89)	1.12 (0.20–2.23)	0.792
*mpo*	1.08 (0.46–1.60)	1.89 (1.13–2.76)	0.336
*mmp-9*	0.90 (0.55–2.05)	1.25 (0.52–1.45)	0.758
*mmp-8*	1.15 (0.44–1.79)	2.22 (0.39–3.09)	0.270

Data are expressed as median (interquartile range).

**Table 3:** Expression of B and T cell markers in spleen and lymph nodes.

Marker (mRNA fold increase)	*apoe*^*−/−*^ (n = 10)	*apoe*^*−/−*^*.Yaa* (n = 8)	p-value
**Spleen**			
**Th1**			
*tim3*	1.13 (1.01–1.65)	2.51 (1.71–3.47)	**0.010**
*ifnγ*	1.99 (0.55–2.27)	0.43 (0.32–1.29)	0.135
**Treg**			
*foxp3*	1.21 (0.97–1.40)	0.87 (0.55–2.41)	0.756
*il10*	1.62 (0.41–1.96)	0.08 (0.02–0.95)	**0.032**
**Th17**			
*rorc*	0.97 (0.58–1.34)	1.36 (0.61–2.63)	0.494
*il17*	0.47 (0.30–1.19)	3.84 (1.18–10.91)	0.072
**Lymph nodes**			
*cd20*	0.95 (0.92–1.07)	1.19 (1.01–1.61)	0.100
*cd4*	0.95 (0.81–1.20)	1.46 (1.19–1.61)	**0.034**
**Th1**			
*tim3*	1.01 (0.87–1.09)	0.87 (0.59–1.32)	0.689
*ifnγ*	1.23 (0.71–1.42)	0.93 (0.61–1.91)	0.961
**Th2**			
*gata3*	0.93 (0.80–1.40)	1.05 (0.87–1.46)	0.625
*il4*	0.99 (0.76–1.21)	0.52 (0.44–0.89)	0.069
**Treg**			
*foxp3*	0.98 (0.71–1.23)	1.10 (0.97–1.25)	0.317
*il10*	1.06 (0.72–1.40)	0.54 (0.45–0.73)	0.083
**Th17**			
*rorc*	1.22 (0.73–1.42)	2.38 (1.40–2.96)	0.056
*il17*	1.12 (0.41–2.39)	0.69 (0.24–0.82)	0.130

Data are expressed as median (interquartile range). Mann–Whitney U test was used for statistical analysis.

Bold values correspond to significant *p* values.

## Discussion

The pathogenesis of atherosclerosis disease is characterized by systemic inflammation. In this context SLE is associated with a significant risk of cardiovascular disease (CVD)^31^. The Framingham Offspring Study showed that the probability of myocardial infarction (MI) matched for age and gender is over 50 times higher in female SLE patients^32^. In this light, we generated an atherosclerosis-lupus-prone Apoe^−/−^Nba2.*Yaa* mouse model to investigate the mechanisms involved in the exacerbation of atherosclerosis in SLE. The present study reveals that atherosclerosis-lupus-prone mice develop vulnerable atherosclerotic plaques in correlation with the production of anti-ApoA-1 IgG and anti-dsDNA IgG antibody levels, both known to have critical pro-atherosclerotic activity^33^.

Macrophages have a key function in the pathogenesis of atherosclerosis by engulfing modified lipoproteins and transforming into lipid-laden foam cells with impaired efferocytosis, leading to necrotic core formation and subsequent plaque destabilization^34^. Pronounced macrophage activation was observed in large cohorts of patients with SLE^35^. Moreover, macrophages in SLE display a defective phagocytic function, enabling an aberrant accumulation of apoptotic debris, leading to the induction of inflammatory responses and breakdown of B cell tolerance^36^. In line with these findings, we found that atherosclerosis-lupus-prone Apoe^−/−^Nba2.*Yaa* mice exhibited increased macrophage accumulation in the aorta. One of the features of the Nba2.*Yaa* lupus-prone mouse is an increase in circulating monocyte counts which could explain the observed increase in plaque macrophages, without affecting plaque size^26^. Consistently, recent publications have shown that more than 50% of foam cells responsible for lipid deposition in the plaque arise from a phenotypical switch of smooth muscle cells^37^. However, the accumulation of macrophages can result in the build-up of uncleared apoptotic bodies, an important source of autoantigens and inflammation which in turn can promote plaque destabilization.

Neutrophils have been shown to have a deleterious effect in atherosclerosis, promoting plaque instability^38,39^. Furthermore, neutrophil extracellular traps (NETs) formation promotes cytotoxic and prothrombotic effects implicated in arterial thrombosis^40,41^, endothelial cell death, lipoprotein modification, and inflammasome activation^42–44^. Moreover, neutrophils from SLE patients exhibit apoptosis and secondary necrosis^45^. Interestingly, we found that neutrophils accumulation in the aortic roots was reduced in Apoe^−/−^Nba2.*Yaa* mice. This could be an effect of neutrophil cell death known to be associated with SLE disease activity and elevated anti-dsDNA antibody levels^46^.

The ruptured plaques are often characterized by a thin and inflamed fibrous cap covering a necrotic core. The matrix metalloproteinases destabilize atherosclerotic plaques through the degradation of elastin, fibronectin, laminin and collagen in correlation with an increased rate of coronary events^47,48^. Apoe^−/−^Nba2.*Yaa* mice exhibited a pronounced increase in MMP-9 expression in the atherosclerotic roots and increased systemic levels of MMP-8 in parallel with a prominent reduction of collagen content in the atherosclerotic roots (Fig. 2). Collagen content is known to be critically important in preventing plaque rupture^49^. The matrix-degrading proteases MMP-8 and MMP-9 degrading components of the extracellular matrix are abundantly expressed in atherosclerotic plaques with a vulnerable histological appearance^47,48^. MMP-9 is a protein expressed and secreted in an inactive form named pro-MMP-9, which is then activated by proteolysis of the propeptide domain^50^. The level of pro-MMP-9 was reduced in the serum of Apoe^−/−^Nba2.*Yaa* mice, indicating an increase in the proteolysis of the pro-peptide MMP-9 in the serum of the Apoe^−/−^Nba2.*Yaa* mice in advanced atherosclerosis. CCL2, which is elevated in Apoe^−/−^Nba2.*Yaa* mice, attracts not only monocytes, but also T lymphocytes and NK cells^51^. Moreover, CCL2 is also known to contribute to plaque destabilization by exerting prothrombotic and inflammatory effects^52^. Taken together, the present findings indicate that plaque vulnerability in atherosclerosis-lupus-prone mouse model is increased, thereby providing a potential explanation for the higher prevalence of CV events in patients with SLE^1,2^.

Anti-ApoA-1 IgG antibodies initially found in SLE patients were associated with a higher prevalence and incidence of CAD with a worse prognosis, independently of traditional CV risk factors observed in autoimmune and non-autoimmune settings^6,12–18^. Anti-ApoA-1 IgG antibodies are described as active modulators of atherothrombosis and linked to a higher incidence of CAD^19–22,53^. Lupus-prone Nba2.*Yaa* mice produce high levels of nucleolar-related autoantibodies and exhibit splenomegaly in addition to a lethal form of lupus nephritis that causes 50% mortality by 14 months of age^24^. In agreement with this finding our atherosclerosis-lupus-prone mouse model showed spleen and lymph node hypertrophy, as well as elevated levels of anti-ApoA-1 antibodies which mediate pro-inflammatory, pro-arrhythmogenic and pro-thrombotic effects^33^. These results could be an indication for a possible mechanism explaining the prominently increased of MI probability in SLE patients^32^. Furthermore, while one feature of atherosclerosis development is the dead cell accumulation leading to genetical material release, the amount of dsDNA correlates with the occurrence of CV events^28^. dsDNA is recognized by the Absent in melanoma 2 (Aim2) inflammasome found in human atherosclerotic lesions in proximity to the necrotic cores^27^, whose activation induces the release of the pro-inflammatory cytokines IL-1β and IL-18^54^. A recent study demonstrated that the dsDNA Aim2 axis triggered a powerful cytokine response in lesional macrophages, thereby enhancing atherosclerotic lesion destabilization^55^. The level of anti-dsDNA IgG in Apoe^−/−^Nba2.*Yaa* mice could therefore be directly associated to unstable plaque vulnerability markers in Apoe^−/−^Nba2.*Yaa* mice on HCD.

T cell response in SLE is very complex, and the Th2 cell polarization observed in Apoe^−/−^Nba2.*Yaa* mice in the present study might be associated with polyclonal B cell activation seen in SLE^56,57^. Moreover, it has been proposed that Th2 and the T_FH_ cytokine IL-4, which is upregulated in the spleen and arch of the Apoe^−/−^Nba2.*Yaa* mice, could rescue B cells from apoptosis and promote autoreactive B lymphocyte survival^58^. Consistently with our present findings, IL-4 treatment triggered the production of anti-dsDNA^58^ antibodies and could thus increase the CD20^+^ B cell population in Apoe^−/−^Nba2.*Yaa* mice on HCD. In SLE murine models, IL-4 knockout mice produced less IgG1 and IgE serum Ig, suggesting a major role of this cytokine in the pathogenesis of the disease^59^. SLE is a systemic autoimmune disease that is known to be associated with polyclonal B cell hyperreactivity with a potential overactive germinal center (GC) and ectopic follicular activity enhancing memory B cells and plasmacytosis^60^. Maturation of the GC and the production of antibodies is dependent on Bcl-6 that is required for follicular T cell differentiation^61^, and Bcl-6 expression was significantly increased in the aortic arch and the spleen of Apoe^−/−^Nba2.*Yaa* mice (Fig. 5). In agreement with this finding, our results indicate that the Nba2.*Yaa* mutation in Apoe^−/−^ mice resulted not only in Th2 cell polarization, but also T_FH_ polarization. Thus, in turn the T_FH_ cells stimulated the induction of CD20^+^ B cells and subsequently elevated anti-dsDNA and apoA-1 IgG antibody levels, leading all together to the exacerbation of atherosclerotic plaque vulnerability in Apoe^−/−^Nba2.*Yaa* mice on HCD. IL-10 is a cytokine known to be essential for regulating the immune response. However, IL-10 also improves B lymphocyte proliferation, Ig class switching and increases antibody secretion^62^. Interestingly, patients with SLE have high levels of IL-10 that are correlated both with the level of disease activity and anti-dsDNA antibody production^63^. In murine models, IL-10 blockade has shown to limit the renal damage and decrease the production of anti-dsDNA antibodies^63^. Although the augmentation of IL-10 was observed in the arch but not in the secondary lymphoid organ in Apoe^−/−^Nba2.*Yaa* mice on HCD, Srikakulapu and colleagues have, however, shown that autoantigen-dependent hypermutation, proliferation, affinity maturation, Ig class switching, memory cell generation, and differentiation into long-lived plasma cells may be carried out in the arterial wall^64^. They also speculate that immature B cells home to artery tertiary lymphoid organs (ATLOs) to undergo differentiation into mature B cells in the absence of the proper control mechanisms acting in the spleen, leading to the production of IgG from autoreactive atherosclerosis and SLE-specific B cells^64^. Altogether, these findings are consistent with the present study, showing a substantial increase of IL-10 expression in the arch of Apoe^−/−^Nba2.*Yaa* mice in parallel with increased levels of CD20^+^ B cells and anti-dsDNA antibodies.

In conclusion, our atherosclerosis-lupus-prone mouse model revealed a mechanism promoting atherosclerotic plaque vulnerability. The Nba2.*Yaa* mutation in Apoe^−/−^ mice was associated with macrophage accumulation, plaque destabilization, the build-up of uncleared apoptotic bodies and subsequent induction of pro-atherogenic antibodies. In parallel, Apoe^−/−^Nba2.*Yaa* mice exhibited atherosclerotic plaque destabilization through a potential Th2 and T_FH_ polarization known to stimulate the induction of CD20^+^ B cells and caused anti-dsDNA and apoA-1 IgG autoantibody production.

## Methods

### Ethical statement

All breeding and experimental protocols and procedures were reviewed and approved by the Institutional Animal Care and Use Committee of the Geneva University School of Medicine. Animal care and experimental procedures were carried out in accordance with the guidelines of the Institutional Animal Care and Use Committee of the Geneva University School of Medicine and complied with the guidelines from Directive 2010/63/EU of the European Parliament on the protection of animals used for scientific purposes.

### Mice

B6.Nba2.*Yaa* mice were generated as described^65^. The Apoe^−/−^ null mutation was introduced in B6.Nba2.*Yaa* mice by breeding. Eleven-week old Apoe^−/−^ C57Bl/6 and Apoe^−/−^Nba2.*Yaa* mice were subjected to 11 weeks of high cholesterol diet (HCD) (20.1% fat, 1.25% cholesterol, Research Diets, Inc., New Brunswick, NJ), as a model of advanced atherosclerosis. The treatments and atherosclerosis protocols were well-tolerated by the mice, and no adverse events (such as weight loss and signs of systemic toxicity) were reported. At sacrifice, haematological parameters, serum triglycerides, total cholesterol, low-density lipoprotein cholesterol (LDL-C), high-density lipoprotein cholesterol (HDL-C), free fatty acids and glucose were routinely measured and expressed in mmol/l. Animals were euthanized by exsanguination after anesthesia with 4% isoflurane. All breeding and experimental protocols and procedures were reviewed and approved by the Institutional Animal Care and Use Committee of the Geneva University School of Medicine. Animal care and experimental procedures were carried out in accordance with the guidelines of the Institutional Animal Care and Use Committee of the Geneva University School of Medicine.

### Quantitative real-time PCR

Total mRNA was prepared by Trizol^®^ (Thermo Fisher Scientific), according to the provider protocol. Reverse transcription was performed using the ImProm-II Reverse Transcription System (Promega, Madison, WI, USA) according to the manufacturer’s instructions. Real-time PCR (StepOne Plus, Applied Biosystems, Waltham, MA, USA) was performed with the SensiFast (LabGene). Real-time duplex qPCR analysis was conducted as described elsewhere^66,67^. The levels of mRNA expression of the target genes was analyzed via real-time PCR using Taqman Gene Expression Assay (Applied Biosystems) for mouse *cd4* (Mm00442754_m1), *gata3* (Mm00484683_m1), *il4* (Mm00445259_m1), *bcl6* (Mm00477633_m1), *cd20* (Mm00545909_m1), *tim3* (Mm00454540_m1), *ifnγ* (Mm01168134_m1)*, foxp3* (Mm00475162_m1), *rorc* (Mm01261022_m1), *il17a* (Mm00439618_m1), *cxcl1* (Mm04207460_m1), *ccl2* (Mm00441242_m1)*, mpo* (Mm01298424_m1)*, mmp-9* (Mm00442991_m1), *mmp-8* (Mm00439509_m1) from Thermo Fisher Scientific. mRNA expression was normalized to the housekeeping gene *hprt* (Mm03024075_m1) using the comparative ΔCT method. All measurements were conducted in triplicate.

### Immunohistochemistry

Mouse aortic sinus was serially cut in 7 μm transversal sections, as previously described^11,68^. Sections from mouse specimens were fixed in acetone and immunostained with specific antibodies anti-mouse CD68 (macrophages, ABD Serotec, Düsseldorf, Germany), anti-mouse Ly-6G (neutrophils, BD PharmingenTM, San Jose, CA, USA), anti-mouse MMP-9 (R&D Systems). Vector Red alkaline phosphatase substrate: SK-5100; in association with Levamisole solution: SP-5000; which produce a magenta coloration is used for revelations (Vector Laboratories, INC, CA, USA). Quantifications were performed using the MetaMorph or Definiens software. Results for other parameters were calculated as percentages of stained area on total lesion area, number of infiltrating cells per mm2 of lesion area or number of lymphatic vessels in adventitia.

### Oil Red O staining for lipid content

Five sections per mouse aortic and aortas sinus were stained with Oil Red O, as previously described^68,69^. Aorta sections were counter-stained with Mayer’s hemalum and rinsed in distilled water. Quantifications were performed using the MetaMorph software. Data were calculated as ratios of stained area on total lesion area.

### Sirius red staining for collagen content

Five sections per mouse aortic sinus were rinsed with water and incubated with 0.1% Sirius red (Sigma Chemical Co, St Louis, MO, USA) in saturated picric acid for 90 min. Sections were rinsed twice with 0.01 M HCl for 1 min and then immersed in water. After dehydration with ethanol for 30 s and cover-slipping, pictures of the sections were taken with ordinary polychromatic microscopy with identical exposure settings. Total collagen content was evaluated under polychromatic light. Quantifications were performed with MetaMorph software. Data were calculated and presented as the percentage of the stained area on total lesion area.

### Serum lipid profile and haematological parameters assessment

Haematology parameters were routinely measured on total blood. Serum lipid and metabolic profiles were routinely measured and expressed as mmol/L. Briefly, blood samples were collected by cardiac puncture, incubated at room temperature for 15 min (for clotting), and the serum was sequentially obtained by centrifugation (4500 rpm for 10 min). The lipids were measured by photometric enzymatic reaction using commercially available kits (glucose, cat. number: #11447513; triglycerides, cat. number: #12016648; total cholesterol, cat. number: #12016630; low-density lipoprotein cholesterol [LDL-c], cat. number: #03038661; high-density lipoprotein cholesterol [LDL-c] cat. number: # 03030024 122; free fatty acids [FFA] cat. number: #11 383 175 001; from Roche Diagnostics GmbH, Mannheim, Germany) using the chemistry analyzer Roche Hitachi 902 (Roche Diagnostics GmbH, Mannheim, Germany). The assay was performed as described in the instruction manual.

### Measurements of serum inflammatory molecule levels

Colorimetric enzyme-linked immunosorbent assay (ELISA) kits to measure serum CCL2, CXCL-1, pro-matrix metalloproteinase (MMP)-9, myeloperoxidase (MPO) and tissue inhibitor of metalloproteinase-1 (TIMP-1) levels (all from R&D Systems), and serum MMP-8 levels (Uscn Life Science Inc., Hubei, China) were used following manufacturer’s instructions. Detection and quantification of the levels of urea nitrogen (BUN) in the serum were performed with Urea Nitrogen Colorimetric Detection Kit (Thermo Fisher Scientific) according to the manufacturer’s instructions.

### Determination of autoantibodies anti-apoA-1 by ELISA

Maxisorp plates (NuncTM, Denmark) were coated with purified, derived delipidated murine recombinant apolipoprotein A-1 (Biorbyt, United Kingdom) (20 mg/ml; 50 ml/well) for 1 h at 37 °C. After washing, all wells were blocked for 1 h with 2% bovine serum albumin (BSA) in phosphate buffer solution (PBS) at 37 °C. Then, samples were incubated for 1 h. Samples were also added to a non-coated well to assess individual non-specific binding. After washing 50 μl/well of signal antibody (alkaline phosphatase-conjugated anti-human IgG; Sigma-Aldrich, St Louis, MO) dilute 1:1000 in PBS/BSA 2% solution was incubated 1 h at 37 °C. After washing, phosphatase substrate p-nitrophanyl phosphate disodium (Sigma-Aldrich) dissolved in diethanolamine buffer (pH 9.8) was added. Each sample was tested in duplicate and absorbance in optical densities (OD) was determined at 405 nm after 20 min of incubation at 37 °C (molecular DevicesTM Filtermax). Corresponding non-specific binding was subtracted from mean absorbance for each sample.

### Determination of autoantibodies anti-dsDNA by ELISA

Salmon Sperm dsDNA was coated to ELISA plates precoated with poly L lysine (Sigma‐Aldrich). Plates were then incubated with 1/100 diluted serum samples, and development performed with alkaline phosphatase‐labelled goat anti‐mouse IgM or IgG. Results are expressed in U/mL in reference to a standard curve.

### Statistical analysis

Statistics were performed using GraphPad Prism 8, Statistica (version 13.0) and the SPSS statistical package (version 20.0). For comparison of two groups of continuous variables, two-tailed unpaired Mann–Whitney *U*-tests with a confidence level of 95% were conducted if data were non-normally distributed. Continuous variables were expressed as median (interquartile range [IQR]) and were compared between the two groups by nonparametric Mann–Whitney U test. Paired intergroup comparisons were performed by using Wilcoxon test. Spearman’s rank correlation coefficients were used to assess correlations between variables. The number of mice used for each analysis is indicated in the figure legends. All data are presented as the mean ± SEM and the statistical significance threshold used is *p ≤ 0.05. **p ≤ 0.05; ***p ≤ 0.005.
